# Multibeam bathymetry data of the western part of the Romanche Trench (Equatorial Atlantic)

**DOI:** 10.1016/j.dib.2021.107198

**Published:** 2021-06-06

**Authors:** Mariia V. Kapustina, Dmitry V. Dorokhov, Vadim V. Sivkov

**Affiliations:** a Shirshov Institute of Oceanology, Russian Academy of Sciences, 36, Nahimovskiy prospekt, Moscow, Russia; b Immanuel Kant Baltic Federal University, Nevskogo Street, 14, 236041 Kaliningrad, Kaliningrad region, Russia

**Keywords:** Multibeam bathymetry, Digital elevation model, Geomorphology, The western part of the Romanche Trench, Equatorial Atlantic

## Abstract

We present the multibeam bathymetry data of the western part of the Romanche Trench (Equatorial Atlantic) which is the main natural corridor that regulates inflow of Antarctic Bottom Water into the eastern basin of the Atlantic Ocean. Multibeam bathymetry survey was carried out during the 33^th^ cruise of the research vessel *Akademik Nikolaj Strakhov* in November 2016. The data were collected using the multibeam echosounder RESON SeaBat 7150 and processed using PDS2000 software. The multibeam bathymetry data are presented as digital elevation models in XYZ tabular format ASCII (*.txt), ESRI ASCII grid (*.asc) and GeoTIFF raster (*.tif) formats with a resolution of 100 m. The dataset is available with the article.

## Specifications Table

**Table:** 

Subject	Seafloor geomorphology
Specific subject area	Multibeam bathymetry
Type of data	Tabular data Digital elevation model (DEM) of the sea bottom relief
How data were acquired	Field survey, shipboard acquisition system. Multibeam echosounder RESON SeaBat 7150, frequency 12.5 kHz.
Data format	Tabular data: ASCII table (∗.txt), DEMs: ESRI ASCII grid (∗.asc), GeoTIFF raster (∗.tif)
Parameters for data collection	Vessel speed 6-8 knots during multibeam survey. The survey was designed as five swaths: - two E-W parallel swaths of ~6.3 nautical miles (nm) (11.6 km) length; - one intersecting S-N swath of ~8.7 nm (16 km) length; - two additional latitudinal swaths: the western one, with length ~2.4 nm (4.5 km) and eastern one (between oceanographic stations) with length ~1.8 nm (3.4 km).
Description of data collection	The raw multibeam data were processed using PDS2000 software (Teledyne Marine). Grid model was created from validated data using PDS2000 and converted into ASCII data, ESRI ASCII grid (*.asc) and GeoTIFF raster (*.tif) using PDS2000 v.3.7.0.47 and QGIS software (v. 3.18.1).
Data source location	The western part of the Romanche Trench (Equatorial Atlantic): Geographical borders of the survey area: 0°59′27″ S - 1°09′10″ S 22°24′12″ W - 22°31′35″ W
Data accessibility	Data are presented with this article

## Value of the Data

• Bottom topography of the deep-sea channels and trenches is needed to clarify the pathways of deep-water masses between the ocean basins.
• The developed DEM represents a high-detailed bathymetry of the western part of the Romanche Trench, including its southern entrance – one of the main sources of the Antarctic Bottom Water (AABW) in Romanche Fracture Zone [1].
• The presented data could be used by hydrologists and geomorphologists for the research planning, sediment cores sampling, CTD profiling and water sampling which require knowledge of the bottom topography.
• The data advance knowledge about modern and past water circulation (AABW).
• The data can contribute to the GEBCO database and can be used in educational programs.

## 1 Data Description

All the data are presented as the supplementary material to the article.

The dataset includes:

- ASCII text file (*.txt) DEM spatial resolution of 100 m in the UTM Zone 27N projection, WGS84 (the structure is presented in Table 1);

**Table 1:** Description of columns in ASCII text table.

Column name	Description
Easting	Easting Cartesian coordinate, UTM Zone 27N projection, WGS84.
Northing	Northing Cartesian coordinate, UTM Zone 27N projection, WGS84.
Depth	Corrected and processed depth, m.
Latitude	Geographical latitude, DD.dd, WGS84.
Longitude	Geographical longitude, DD.dd, WGS84.
- ESRI ASCII grid (*.asc) DEM spatial resolution of 100 m in the UTM Zone 27N projection, WGS84;
- GeoTiff raster (*.tif) of DEM spatial resolution of 100 m in the UTM Zone 27N projection, WGS84;
- Two figures (*.png). Fig. 1 shows the study area, Fig. 2 shows the developed DEM.

**Fig. 1:**
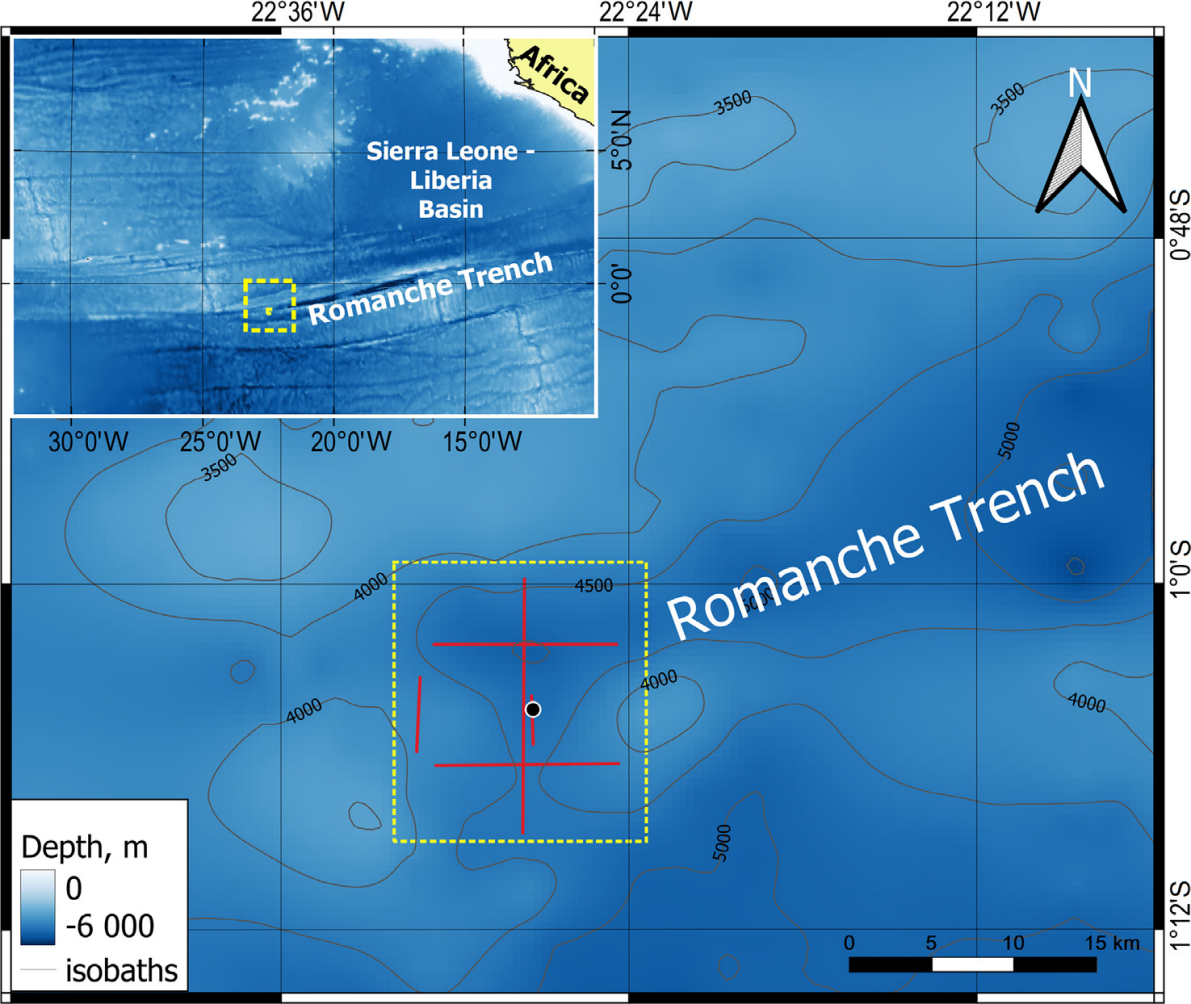
Study area investigated during the 33^th^ cruise of the research vessel *Akademik Nikolaj Strakhov*. Red lines – survey lines, yellow dotted rectangle – survey area, black circle – station 17795295 used for SVP. Bathymetry is based on the [4].

**Fig. 2:**
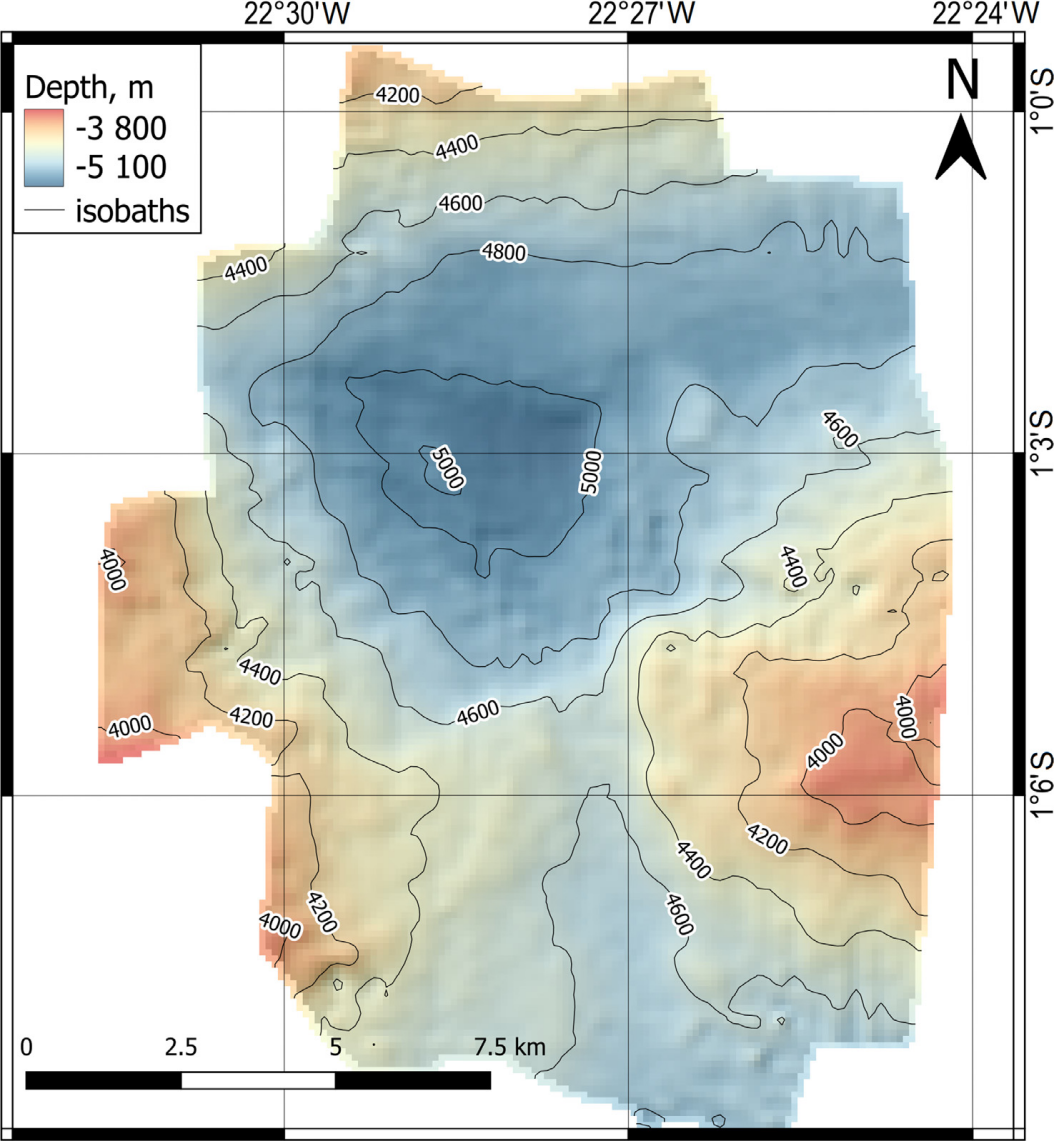
DEM of the western part of the Romanche Trench.

Provided data are fully processed.

## 2 Experimental Design, Materials and Methods

Multibeam bathymetry survey was carried out at the western part of the Romanche Trench during the 33^th^ cruise of the research vessel *Akademik Nikolaj Strakhov* in 22^nd^ of November 2016 [2] (Fig. 1). The survey was designed as five swaths: two E-W parallel swaths, one intersecting and two additional latitudinal swaths; the vessel speed was 6–8 knots. Single swath of MBE covers 8–10 km. The total length of survey lines was 19.2 nm. Total surveyed area was about 181 km^2^.

The data were collected using the system RESON SeaBat 7150 (12.5 kHz, 256 beams) with an integrated navigation system Applanix POS MV. There are no tidal stations in the study area, besides in accordance with the model data tidal range within the survey area constitutes a relatively small. Tidal corrections were ignored. Errors for each log file were obtained with the export utility in PDS2000. Vertical error was 2.05±2.06 m, horizontal error was 23.2±20.8 m. The multibeam echosounder calibration (Patch Test) was performed.

Data collection and processing were carried out using the PDS2000 software v.3.7.0.47. Data processing consisted of several stages: applying the sound velocity profile correction (SVP-correction), input calibration corrections, removing outliers with filters, manual rejection of the errors, creating a grid model, and exporting the data in ASCII and ASCII ESRI formats. SVP was calculated from temperature, salinity, and pressure. Due to the absence of measurements of the sound velocity profile in the study area, for the SVP corrections we used the data from WOD2019 [3] obtained in September 2016 at the station 17795295 (Fig. 1). The maximum depth of the SVP used to correct the multibeam data was 4896 m. SVP was imported to PDS2000 using the «Sound Velocity Profile Editor».

The spatial resolution of DEM is 100 × 100 m and presented in UTM Zone 27N projection, datum WGS84 (Fig. 2).

## CRediT Author Statement

**Mariia V. Kapustina:** Data curation, Visualization, Writing - original draft; **Dmitry D. Dorokhov:** Data curation, Validation; **Vadim V. Sivkov:** Conceptualization, Funding acquisition, Supervision.

## Declaration of Competing Interest

The authors declare that they have no known competing financial interests or personal relationships which have, or could be perceived to have, influenced the work reported in this article.

## Appendix Supplementary materials

Image, application 1

Image, application 2
